# Establishing denominator-consistent Six Sigma benchmarks for venous blood specimen rejection: a multi-center retrospective quality-indicator study using 21 million specimens in Western China

**DOI:** 10.3389/fmed.2026.1878028

**Published:** 2026-08-17

**Authors:** Chunbao Xie, Changrui Sun, Li Xiao, Tao Chen, Fengxi Jia, Fangkui Xiong, Zehong Wang, Yulian Zhou, Hengyong Yu, Huan Ding, Yifeng Huang, Xiaoting Zhan, Guangcheng Luo, Jiawei Zeng, Yi Huang

**Affiliations:** 1 Department of Laboratory Medicine, Sichuan Provincial People’s Hospital, University of Electronic Science and Technology of China, Chengdu, Sichuan, China; 2 Sichuan Provincial Key Laboratory for Human Disease Gene Study, Chengdu, Sichuan, China; 3 Department of Clinical Laboratory, Zigong First People’s Hospital, Zigong, Sichuan, China; 4 Department of Clinical Laboratory, Ya’an People’s Hospital, Yaan, Sichuan, China; 5 Department of Laboratory Medicine, Liangshan Prefecture People’s Hospital, Xichang, Sichuan, China; 6 Medical Laboratory, Jinniu District People’s Hospital of Chengdu, Chengdu, Sichuan, China; 7 Department of Laboratory Medicine, Medical Center Hospital of Qionglai City, Chengdu, Sichuan, China; 8 Department of Clinical Laboratory, Mianyang Central Hospital, School of Medicine, University of Electronic Science and Technology of China, Mianyang, Sichuan, China; 9 Department of Clinical Laboratory, Qianjiang District TCM Hospital of Chongqing, Chongqing, China; 10 Depariment of Clinical Laboratory, Zhongxian People’s Hospital of Chongqing, Chongqing, China; 11 Chengdu Wenjiang District People's Hospital, Chengdu, Sichuan, China; 12 Department of Medical Laboratory, The Affiliated Hospital of Southwest Medical University, Luzhou, Sichuan, China; 13 Department of Clinical Laboratory Medicine, Affiliated Hospital of North Sichuan Medical College, Nanchong, Sichuan, China; 14 Medical Administration Department, Sichuan Academy of Medical Sciences and Sichuan Provincial People’s Hospital, Chengdu, Sichuan, China

**Keywords:** laboratory medicine, multi-center study, pre-analytical error, quality benchmarking, quality indicators, Six Sigma, specimen rejection

## Abstract

**Background:**

Pre-analytical venous blood specimen rejection can delay clinical decision-making and may increase laboratory workload, yet multicenter benchmarking remains limited by inconsistent rejection definitions and denominator selection. This study aimed to support denominator-consistent benchmarking, transferable implementation, and quality monitoring across heterogeneous-resource settings.

**Methods:**

We conducted a retrospective quality-indicator study across 10 hospitals in Western China using routine venous blood specimen data collected from January 1, 2023, to April 30, 2024. Rejection reasons were standardized with a prespecified mapping dictionary. Center-level rejection rates, defects per million opportunities (DPMO), and Six Sigma values were calculated using monthly venous specimen volume as the primary denominator. Percentile-based benchmarks, pooled estimates, and a month-adjusted random-intercept generalized linear mixed-effects model were used to assess performance and residual between-center heterogeneity.

**Results:**

Among 21,319,200 venous specimens, 42,060 were rejected, yielding a pooled rejection rate of 0.197%. The leading standardized rejection categories were clot/aggregation, insufficient or incorrect volume, and container/tube error. Rejection patterns varied across centers and clinical settings: inpatient and emergency events were dominated by clotting and volume problems, whereas outpatient and physical examination events more often showed order, timing, or identification errors. After temporal adjustment, measurable between-center heterogeneity persisted (intraclass correlation coefficient = 0.045).

**Conclusion:**

This multi-center quality-indicator study provides a reproducible, denominator-consistent framework for benchmarking venous blood specimen rejection across multiple centers. Percentile-based benchmarks and standardized reason spectra may support local implementation and transferable Six Sigma monitoring in healthcare systems with heterogeneous resources.

## Introduction

Pre-analytical errors account for a large proportion of laboratory quality failures (1–4). Specimen rejection represents a measurable and actionable quality indicator (5, 6). Rejection events can delay diagnosis and treatment, increase costs, and burden both laboratory and clinical services (7–9).

Despite widespread use of rejection rates as quality indicators, “benchmarking” across multiple centers remains challenging (10, 11). Differences in operational definitions (what constitutes a reportable rejection event), reason taxonomies, and denominator construction can lead to benchmarks that are not reproducible or transferable across settings.

In this context, regional multi-center analyses have value beyond describing local performance. When implemented with reproducible definitions and denominator consistency, they enable: (1) estimation of center-level performance distributions that are less dominated by high-volume centers than pooled estimates, and (2) construction of practical benchmarks (e.g., percentile-based targets and Six Sigma capability) that can be applied for system-level quality management. While this study is based on data from Western China, the analytical framework and standardized definitions are designed to be adaptable to other healthcare systems with comparable laboratory information infrastructure, provided that local definitions and denominators are harmonized accordingly.

This study aimed to provide a reproducible benchmarking framework for venous blood specimen rejection by characterizing: (i) center-level rejection rates and Six Sigma capability (12, 13) using a prespecified venous_total denominator, (ii) a standardized rejection reason spectrum based on a frozen dictionary, (iii) distribution-based benchmarks for operational use, (iv) stratified rejection reason patterns across centers, encounter settings, and standardized specialty departments, (v) between-center heterogeneity after accounting for temporal variation, and (vi) practical implementation requirements for denominator-consistent benchmarking in heterogeneous-resource settings.

## Methods

### Study design and setting

This was a multi-center, retrospective observational study in a laboratory quality-indicator framework. The analysis period for the main study was 2023-01-01 to 2024-04-30.

### Data sources

Each center exported a rejection-event log and monthly denominator sheets from the laboratory information system (LIS) or pre-analytical quality-management system. Because the participating centers used heterogeneous local systems and field configurations, there was no single uniform LIS query string applicable to all centers. Instead, source workbooks were checked for the prespecified event-field set (date, specimen type, tube label, department, rejection reason, and test item) and matching monthly venous_total denominators. Center-level field availability, submitted-month coverage, denominator QC, and department-denominator coverage are reported in Supplementary Table S13. No center-level rejection rate was extrapolated beyond the submitted denominator months.

### Eligibility criteria

#### Main analysis

Included events were restricted to routine venous blood specimens (whole blood/serum/plasma) in the prespecified time window. The main analysis denominator was each center’s monthly venous specimen volume (venous_total).

#### Exclusions

Non-venous specimens and unclassifiable specimen types were excluded from the main analysis. Blood culture contamination events were excluded from the main analysis and analyzed as a prespecified Supplementary dataset.

### Variables and standardization

#### Core variables

For each event, the standardized dataset includes: center_id, date and ym (YYYY-MM), dept_raw, specimen_raw, specimen_class, tube_raw, reason_raw.

#### Reason mapping

Rejection reasons were cleaned by normalizing whitespace and punctuation and by removing suffixes indicating rate-based reporting before mapping to standardized primary categories (reason_std_primary) using a frozen dictionary (Supplementary Table S1). The dictionary was developed iteratively: an initial categorization was derived from exploratory analysis of the pooled free-text reason entries across all centers, then consolidated through group discussion among co-investigators and refined over multiple rounds until no new categories emerged. The final dictionary was frozen prior to quantitative analysis and is provided in full as Supplementary Table S1 to enable independent auditing and adaptation to other settings.

### Department classification

Department-derived subgrouping was handled at two levels. First, because encounter setting was not available as a dedicated structured field, a conservative explicit-label approach was used for encounter-setting summaries: records were classified as inpatient, outpatient, emergency, or physical examination only when the raw department label itself contained an explicit setting indicator such as outpatient, inpatient ward, emergency, physical examination, or health management; all remaining records were retained in the main dataset but treated as unclassified for encounter-setting analyses. Second, for specialty-oriented summaries, raw department labels were standardized into mutually exclusive specialty groups using a prespecified priority-ordered rule table iteratively expanded during audit of high-frequency raw labels (e.g., ICU/critical care, pediatrics/neonatal, respiratory, hematology/oncology, gastroenterology/hepatobiliary, nephrology/rheumatology, and neurology/neurosurgery). This distinction was important because the absence of a structured patient-type field constrained encounter-setting assignment more severely than specialty classification.

#### Tube mapping

Tube categories were mapped from local tube labels to standardized proxy groups using a rule table. Additive information was not uniformly available in the exported fields; consequently, color-derived categories represent locally recorded tube-label proxies rather than verified additive-specific tube classes. Center-level differences in these proxy distributions are reported in Supplementary Table S12, and all tube analyses are reported as rejection-event compositions rather than tube-specific rejection rates.

#### Outcomes

The primary outcome was the center-level venous specimen rejection rate *p* = D/N, where D is the number of venous rejection events and N is the venous_total denominator.

Secondary outcomes included defects per million opportunities (DPMO) and Six Sigma capability. Sigma was calculated using the conventional 1.5-Sigma shift (12).


$$\text{DPMO}=\frac{D}{N}\times 10^{6}$$

$$\sigma =\Phi^{-1}(1-p)+1.5,\;\;p=\frac{D}{N}$$

### Statistical analysis

Categorical variables are presented as *n* (%). Proportion 95% confidence intervals were computed using Wilson score intervals (14).

For benchmarking, results are presented primarily as the distribution of center-level rejection rates (median, IQR, P10–P90), supplemented by a pooled (volume-weighted) estimate. Volume-weighted pooling was used for the overall summary because it reflects the actual clinical burden distribution; however, it is dominated by the highest-volume center and is therefore presented only as a secondary benchmark descriptor, with the center-level distribution as the primary reference.

A binomial, month-adjusted random-intercept generalized linear mixed-effects model (GLMM) was fitted on center-month aggregated data:


$$D_{\mathrm{cm}}∼\text{Binomial}(N_{\mathrm{cm}},p_{\mathrm{cm}})$$

$$\text{logit}(p_{\mathrm{cm}})=\beta_{0}+u_{c}+\sum_{m\in M}\beta_{1m}I(ym_{\mathrm{cm}}=m)$$

Where u_c is a center-specific random intercept and month is treated as a categorical fixed effect with 15 dummy indicators (reference: 2023-01), capturing any non-linear temporal variation in monthly rejection probability. The final reporting model was cbind(D, *N* − D) ~ ym + (1 | center_id); hospital grade was not included as a model predictor and was summarized descriptively in Table 1. The center random-intercept variance and ICC on the logistic scale were reported to quantify between-center heterogeneity.


$$\mathrm{ICC}=\frac{\tau^{2}}{\tau^{2}+\pi^{2}/3}$$

**Table 1:** Center-level quality indicators for venous specimen rejection (main analysis).

Center	Hospital level	Months	N (venous_total)	D (rejections)	Rejection rate (Wilson 95% CI)	DPMO	Sigma (1.5 shift)
004	Tertiary grade A (3A)	16	7,806,713	23,421	0.300% (0.296–0.304%)	3000.1	4.248
005	Tertiary grade A (3A)	16	3,062,426	4,498	0.147% (0.143–0.151%)	1468.8	4.474
018	Tertiary grade B (3B)	16	918,396	1,304	0.142% (0.134–0.150%)	1419.9	4.485
030	Tertiary grade A (3A)	16	1,561,199	2,721	0.174% (0.168–0.181%)	1742.9	4.421
031	Tertiary grade A (3A)	16	926,318	1,697	0.183% (0.175–0.192%)	1832.0	4.406
035	Secondary grade A (2A)	16	244,149	165	0.068% (0.058–0.079%)	675.8	4.705
CB	Tertiary grade A (3A)	16	2,692,558	3,995	0.148% (0.144–0.153%)	1483.7	4.471
LS	Tertiary grade A (3A)	16	1,250,185	1,602	0.128% (0.122–0.135%)	1281.4	4.516
YA	Tertiary grade A (3A)	16	1,204,237	1,098	0.091% (0.086–0.097%)	911.8	4.618
ZG	Tertiary grade A (3A)	12	1,653,019	1,559	0.094% (0.090–0.099%)	943.1	4.608
Pooled	All centers (volume-weighted)	NA	21,319,200	42,060	0.197% (0.195–0.199%)	1972.9	4.382

*N* is the monthly venous specimen denominator (venous_total) aggregated across the study period. Rejection rate confidence intervals were computed using Wilson score intervals. Sigma was computed as inverse normal (1 − p) + 1.5, where *p* = D/N.

For analyses requiring department denominators, department-level rejection rates were computed only for center datasets passing a denominator harmonization check. Specifically, the coverage ratio was defined as:


$$\text{Coverage ratio}=\frac{\sum_{d}N_{\mathrm{cd}}}{N_{c}}$$

Department-level rates were reported only when coverage_ratio fell within the prespecified range [0.98, 1.02]; centers outside this range were excluded from department-level rate inference and were used only for denominator-free summaries (e.g., event compositions). This restriction improves internal validity of department-level estimates but limits external validity/generalizability of department-level rate findings to centers with harmonized department denominators (and may exclude high-volume centers).

A sensitivity analysis restricted to months common across all centers (2023-05 to 2024-04) was prepared to assess robustness to variable time coverage.

Differences in standardized reason distributions across centers, explicit encounter settings, and standardized specialty departments were evaluated using contingency-table chi-square tests.

Data preparation and table generation were performed in Python 3.12.13 using pandas 2.2.3, openpyxl, and xlrd. Wilson score intervals were implemented from the published formula (14) using Python standard-library routines, and contingency-table chi-square tests used SciPy. The center-month generalized linear mixed-effects model was fitted in R 4.6.0 using lme4 2.0.1. All tests were two-sided with alpha = 0.05.

Multiple comparisons, when performed, used Benjamini–Hochberg FDR control.

The contextual published-rate comparison was retained only as narrative context and was not treated as a systematic review or meta-analysis.

### Pareto concentration analysis (Top-2)

To summarize heterogeneity in rejection reason spectra in an operationally interpretable way, we quantified within-center Pareto concentration using the Top-2 cumulative share. For each center c, let n_{c,k} denote the number of venous rejection events mapped to standardized primary reason category k (reason_std_primary). We ranked categories within each center by descending n_{c,k} and defined:


$$\mathrm{Top}-2\;{\text{share}}_{c}=\frac{n_{c,(1)}+n_{c,(2)}}{\sum_{k}n_{c,k}}$$

Where (1) and (2) denote the first and second most frequent categories within center c. All standardized primary categories were eligible, including Other/Unknown, consistent with reporting in Table 2; ties in counts were resolved deterministically by ordering tied categories alphabetically by category label—a transparent convention that avoids arbitrary manual assignment while acknowledging that tied categories have equivalent statistical priority. Across centers, Top2Share_c was summarized using the median and the 10th to 90th percentile range (P10–P90).

**Table 2:** Standardized rejection reason spectrum (main analysis; venous specimens).

Reason (standardized primary)	*n*	% of venous rejections
Clot/Aggregation	11,322	26.92%
Insufficient/Incorrect volume	9,548	22.70%
Container/Tube error	6,292	14.96%
Order error	3,758	8.93%
Other/Unknown	2,325	5.53%
Label/Identification error	2,262	5.38%
Hemolysis	2,208	5.25%
Timing error	2,198	5.23%
Specimen type error	1,316	3.13%
Transport time	821	1.95%
Transport temperature	10	0.02%

The center-specific standardized reason distribution is shown as a heatmap in Figure 2, and the full long-format numeric values are provided in Supplementary Table S7. Reasons are standardized using a frozen mapping dictionary (Supplementary Table S1). Percentages in Table 2 are based on the total venous rejection events (*n* = 42,060). Cell values are within-center percentages of venous rejection events, not percentages of the total pooled denominator. Overall percentages among all venous rejection events are shown in Table 2. The Other/Unknown category showed substantial between-center variation (range: 0.04–31.37%), reflecting differences in the completeness and specificity of rejection reason documentation across sites rather than differences in actual rejection etiology; centers with high Other/Unknown proportions should be interpreted with caution.

### Ethics

This retrospective, multi-center quality-indicator study used secondary data extracted from hospital laboratory information systems and quality-management exports. The protocol was reviewed and approved by the Ethics Committee of Sichuan Provincial People’s Hospital (approval number: 2024205), and the requirement for informed consent was waived because the analysis used minimal-risk, de-identified operational data generated during routine care and quality management.

De-identification was implemented before analysis by removing direct personal identifiers. Data were limited to operational variables required for quality-indicator benchmarking, and no patient-level clinical outcomes were accessed. Access to source extracts and analytic datasets was restricted to authorized study personnel under applicable institutional data-governance procedures. This manuscript does not claim separate ethics approvals at every participating center beyond the documented coordinating-center approval.

### Data governance, reproducibility, and availability

Source data were derived from clinical laboratory systems and are subject to institutional and regulatory restrictions; therefore, patient-level and event-level raw extracts cannot be publicly shared. The frozen rejection-reason dictionary, aggregated benchmark outputs, and figure-ready summary datasets may be shared upon reasonable request to the corresponding author, subject to institutional approval and any required data-use agreement.

## Results

### Study flow and dataset construction

**Figure 1:**
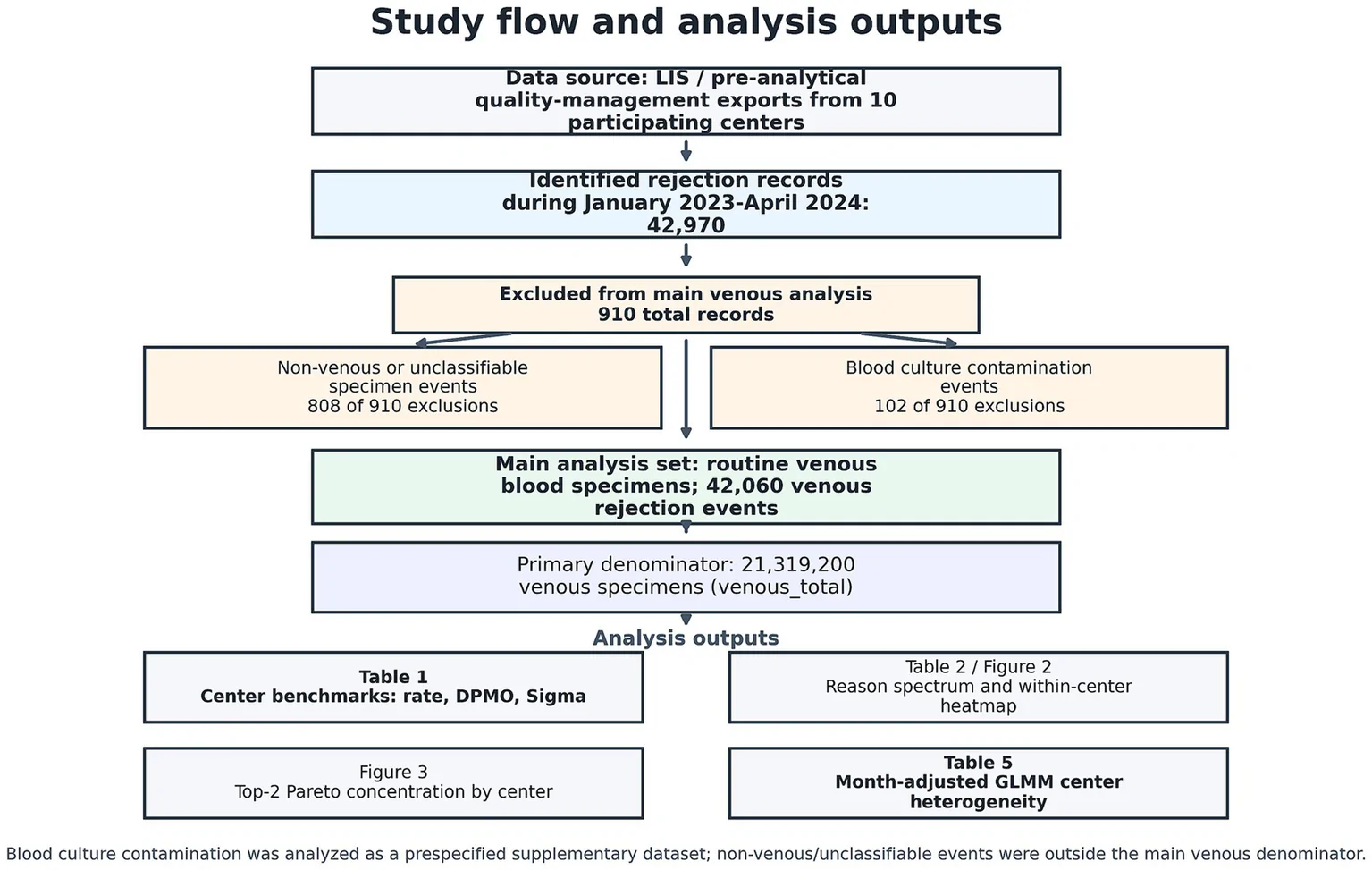
Study flow and analysis outputs for the main venous rejection analysis. The flowchart summarizes LIS/pre-analytical quality-management data sources from 10 centers, prespecified exclusions, the 42,060 venous rejection-event main analysis set, the 21,319,200 venous_total denominator, and the downstream table, figure, and model outputs.

A total of 42,970 rejection records were identified in the study window. After prespecified exclusions for the main analysis (total excluded *n* = 910: 808 non-venous or unclassifiable specimen events [1.88%] and 102 blood culture contamination events [0.24%]), the main analysis included 42,060 venous rejection events. The study flow and downstream analysis outputs are summarized in Figure 1. Blood culture contamination events (*n* = 102) occurred predominantly in two centers (CB: *n* = 83; ZG: *n* = 15; 004: *n* = 4); their distribution by center and local reason label is summarized in Supplementary Tables S2 and S3.

### Center-level quality indicators

Center-level QI metrics (N, D, rejection rate with Wilson 95% CI, DPMO, and Sigma) are provided in Table 1.

Sensitivity analyses assessed variable time coverage, influential centers, and conservative duplicate-pattern removal. In the common-month window, the pooled rejection rate was 0.192% and P10 was 0.067%, compared with 0.197 and 0.089% in the full window (Supplementary Table S4). Excluding the highest-volume center (004) reduced the full-window pooled rate to 0.138% (Supplementary Table S5). Under strict removal of records identical on center, date, department, specimen type, tube label, and rejection reason, 6,866 records were removed and the pooled rate decreased to 0.165% (Supplementary Table S14). These analyses support stability of the leading reason ranking but show that absolute pooled rates and lower-tail benchmark percentiles remain sensitive to coverage, influential centers, and possible duplicate logging.

### Rejection reason spectrum

**Figure 2:**
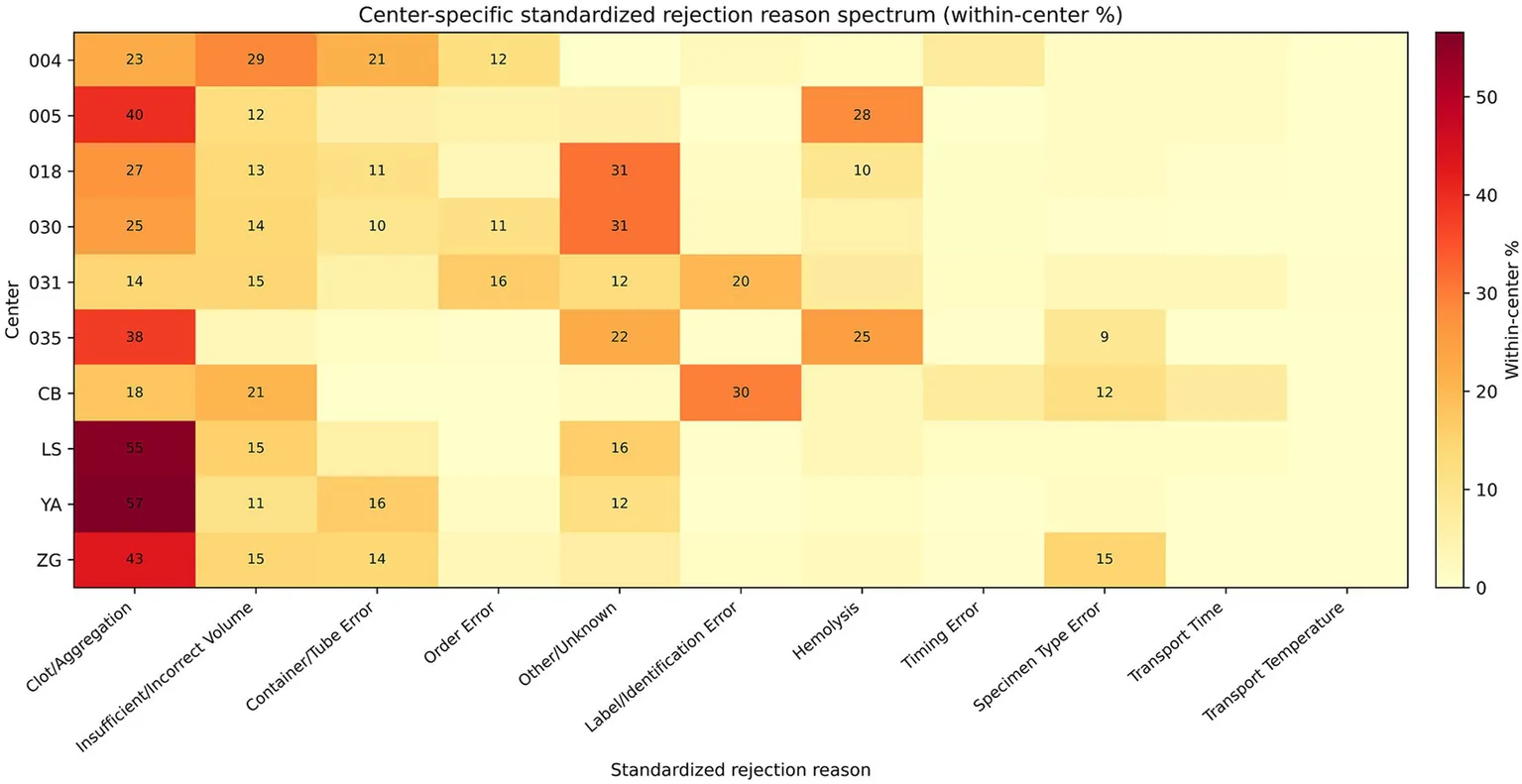
Heatmap of center-specific standardized rejection reason spectrum (within-center %). Rows represent centers and columns represent standardized primary rejection reasons. Cell colors encode the within-center percentage for each reason category, with darker cells indicating higher within-center prevalence. Cell values are within-center percentages of venous rejection events, not percentages of the total pooled denominator. Overall percentages among all venous rejection events are shown in Table 2. The full numeric long-format table is provided in Supplementary Table S7.

Overall and center-specific standardized reason spectra are provided in Table 2 and Figure 2. Cell values are within-center percentages of venous rejection events, not percentages of the total pooled denominator. Overall percentages among all venous rejection events are shown in Table 2. Between-center reason distributions differed significantly (Chi-square = 27,668.3; df = 90; *p* < 0.001). At the pooled level, the Top-3 reason set remained unchanged after excluding Other/Unknown (Supplementary Table S6); because removing one category necessarily preserves the relative ordering of the remaining categories, this result is not interpreted as a center-level Pareto stability test.

The full long-format center-specific numeric values are provided in Supplementary Table S7.

**Figure 3:**
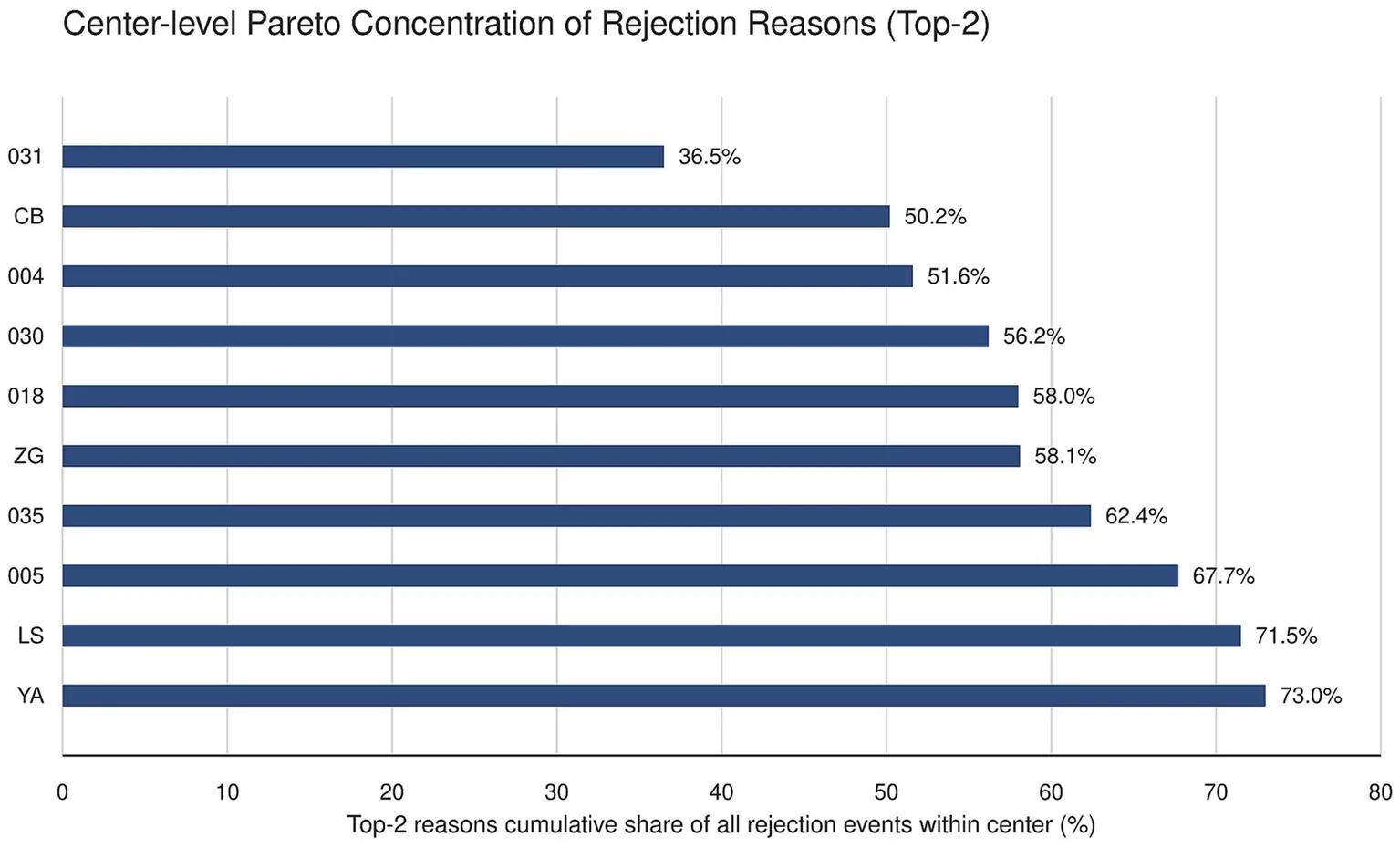
Center-level Pareto concentration of rejection reasons (Top-2). Top-2 is defined as the two most frequent standardized rejection reasons within each center. The primary bars include Other/Unknown when it is among the two most frequent categories; Supplementary Table S11 provides the targeted center-level sensitivity analysis after excluding Other/Unknown.

Figure 3 summarizes the cumulative share of total rejection events explained by the two most frequent standardized reason categories within each center (Top-2), including Other/Unknown when it was among the two most frequent categories. Across centers, the original Top-2 cumulative share had a median of 58.0% (P10-P90, 48.9–71.6%). In a targeted sensitivity analysis excluding Other/Unknown within each center, the median was 60.3% (P10-P90, 50.1–83.0%), and the Top-2 category composition changed in centers 018, 030, and LS (Supplementary Table S11).

### Encounter-setting and standardized-department stratified patterns

Among the 42,060 venous rejection events, 15,757 (37.5%) carried explicit encounter-setting labels and were eligible for conservative subgroup summaries: inpatient, *n* = 8,483; outpatient, *n* = 4,491; emergency, *n* = 2,392; and physical examination, *n* = 391. The encounter-setting by reason distribution differed significantly (Chi-square = 3,375.5; df = 30; *p* < 0.001). Complete eligible-setting sample sizes and the three leading reason counts and within-setting percentages are reported in Supplementary Table S9. Inpatient and emergency events were dominated by clot/aggregation and volume-related categories, whereas outpatient and physical-examination records showed larger contributions from order, timing, and label/identification categories.

After specialty standardization of raw department labels, 40,643/42,060 events (96.6%) could be assigned to one of 26 department groups, and the standardized-department by reason distribution also differed significantly (chi-square = 6,426.1; df = 250; p < 0.001). ICU/critical care events were dominated by Clot/Aggregation (2,811/7,114, 39.5%) and Insufficient/Incorrect Volume (1,666/7,114, 23.4%). Pediatrics/neonatal departments showed a similar pattern, with Clot/Aggregation accounting for 42.5%, Insufficient/Incorrect Volume for 23.6%, and Hemolysis for 11.0%. Respiratory departments displayed a more even three-way distribution among Container/Tube Error (28.2%), Insufficient/Incorrect Volume (24.5%), and Clot/Aggregation (16.8%), whereas hematology/oncology departments remained primarily driven by Clot/Aggregation (25.6%), Insufficient/Incorrect Volume (20.3%), and Container/Tube Error (17.7%).

### Department distribution

Department-category event composition is reported without denominators. Seven centers passed the prespecified department-denominator coverage QC; their center-level coverage results and available department-rate ranges are reported in Supplementary Table S10, and the full filtered center-department rate estimates with Wilson 95% confidence intervals are provided in Supplementary Data S1. Centers 004, 031, and ZG did not pass the coverage criterion and were excluded from department-rate inference.

The standardized specialty-department composition of rejection events is summarized in Table 3.

**Table 3:** Standardized specialty-department composition of venous rejection events (denominator-free; all centers).

Standardized department	*n*	% of venous rejections
ICU/Critical care	7,114	16.91%
Gastroenterology/Hepatobiliary	4,233	10.06%
Pediatrics/Neonatal	3,074	7.31%
Nephrology/Rheumatology	3,030	7.20%
Respiratory	2,648	6.30%
Neurology/Neurosurgery	2,629	6.25%
Cardiology/Cardiovascular	2,433	5.78%
Hematology/Oncology	2,351	5.59%
Orthopedics/Burn/Rehabilitation	1,960	4.66%
General surgery	1,561	3.71%
Other standardized groups combined	9,610	22.85%
Unclassified	1,417	3.37%

This table reports the distribution of rejection events by standardized specialty department and does not require department denominators. Standardization was based on a priority-ordered rule table applied to raw department labels; 40,643/42,060 events (96.6%) were classified, leaving 1,417 (3.4%) unclassified, mostly under generic labels such as outpatient registration or self-service order clinic that were not suitable for forced specialty assignment. This table should be interpreted as a descriptive specialty-composition summary rather than as a department-specific rate benchmark.

### Tube and specimen composition

Tube-label proxy and specimen-class results are reported as rejection-event distributions. Supplementary Table S12 shows the center-level distribution of locally recorded tube-label proxy groups; these categories should not be interpreted as verified additive-specific tube classes.

The distribution of venous rejection events by locally recorded tube-label proxy group and specimen class is summarized in Table 4.

**Table 4:** Distribution of venous rejection events by locally recorded tube-label proxy group and specimen class (denominator-free; all centers).

Analysis dimension	Local tube-label group / specimen class	*n*	% of venous rejections
Tube-label proxy	Purple label	13,702	32.58%
Tube-label proxy	Blue label	11,402	27.11%
Tube-label proxy	Red label	9,193	21.86%
Tube-label proxy	Green label	3,672	8.73%
Tube-label proxy	Yellow label	2,492	5.92%
Tube-label proxy	Other tube labels	866	2.06%
Tube-label proxy	Gray label	282	0.67%
Tube-label proxy	Syringe	172	0.41%
Tube-label proxy	Blood gas syringe	141	0.34%
Tube-label proxy	Other/Unknown	86	0.20%
Tube-label proxy	Blood culture bottle	52	0.12%
Specimen class	Whole blood	17,768	42.24%
Specimen class	Plasma	13,164	31.30%
Specimen class	Serum	11,128	26.46%

Tube-label proxy and specimen results are presented as rejection-event compositions because tube-specific and specimen-specific denominators were unavailable. Color-derived categories are locally recorded proxy labels rather than verified additive-specific tube classes; center-level proxy distributions are shown in Supplementary Table S12.

### Between-center heterogeneity after temporal adjustment

After re-verification of hospital accreditation, the final hospital-grade distribution was heavily imbalanced (3A, *n* = 8 centers; 3B, *n* = 1; 2A, *n* = 1). We therefore retained hospital grade as a descriptive center characteristic in Table 1 and focused formal inference on residual between-center heterogeneity after temporal adjustment. In the month-adjusted random-intercept GLMM without hospital grade as a predictor, the center random-intercept variance was 0.155 (SD 0.393; ICC = 0.045), indicating measurable residual variation in rejection probability across centers beyond calendar-month effects. The model summary is reported in Table 5.

**Table 5:** Summary of between-center heterogeneity after temporal adjustment in the center-month GLMM.

Parameter	Value	Additional information
Fixed intercept (beta)	−6.368	SE 0.126
Fixed intercept OR	0.0017	95% CI 0.0013–0.0022; *p* < 0.001
Random intercept variance (center)	0.155	SD 0.393
ICC	0.045	Logistic-scale ICC
Centers	10	Included in the final GLMM
Months	16	Calendar months in the full study window
Center-month rows	156	Aggregated observations

The GLMM was fitted on center-month aggregated data with a random intercept for center and month fixed effects. Hospital grade was summarized descriptively in Table 1 and was not included as a model predictor. ICC was reported on the logistic scale as tau^2/(tau^2 + pi^2/3).

## Discussion

This retrospective quality-indicator study presents a reproducible, denominator-consistent framework for multi-center benchmarking of venous blood specimen rejection. Center-level distributions are preferable to relying only on pooled estimates because volume-weighted pooled rates can be dominated by the largest centers and may obscure variation relevant to local quality management. Prioritizing the median, IQR, and P10–P90 of center-level rates provides a more interpretable reference for hospitals seeking to compare their own performance against a regional distribution.

The encounter-setting patterns suggest different operational priorities across care contexts without establishing causal workflow drivers. Inpatient and emergency records were dominated by clot/aggregation and volume-related categories, which may indicate a need to review venipuncture, anticoagulant handling, and specimen mixing workflows in high-acuity settings. Outpatient and physical-examination records showed larger contributions from order, timing, and label/identification categories, pointing instead to front-end workflow coordination, identity verification, and pre-collection preparation as practical review targets.

The global and national comparisons should be interpreted as contextual only. Published rejection rates differ in specimen scope, laboratory section, acceptance criteria, denominators, and reporting practice; therefore, direct numerical comparisons across international programs, Chinese monitoring programs, and single-center reports should not be treated as equivalent benchmark targets (13, 15).

High Other/Unknown proportions reduce the actionability of Pareto concentration benchmarks because dominant categories may reflect incomplete documentation rather than specific intervention targets. The targeted center-level sensitivity analysis changed the Top-2 category composition in centers 018, 030, and LS and increased the cohort median Top-2 share from 58.0 to 60.3% after excluding Other/Unknown (Supplementary Table S11). Pareto values for centers with high Other/Unknown proportions should therefore be interpreted together with documentation completeness and the sensitivity result.

At minimum, hospitals implementing this framework should capture an event-level rejection log containing center identifier, date or month, department or encounter-setting label, specimen type, tube or additive label, raw rejection reason, and a monthly denominator file containing venous_total for the same time window. Optional fields such as specimen barcode, tube-specific denominators, and department-level venous totals would strengthen deduplication and stratified rate analyses.

This framework is transferable only when local definitions and denominators are harmonized. The frozen dictionary should be adapted to local terminology before use. Sites with incomplete reason documentation should first improve coding specificity before relying on Pareto concentration for intervention selection.

The center-level benchmark approach is therefore best viewed as a practical implementation framework rather than a universal external standard.

Sensitivity analyses showed different degrees of stability across estimands. The leading standardized reason categories were stable, but the pooled absolute rate and lower-tail percentiles changed with common-month restriction, exclusion of the highest-volume center, and strict duplicate-pattern removal (Supplementary Tables S4, S5, S14). The cohort-derived percentiles are therefore descriptive internal references rather than universal or externally validated performance thresholds.

Improving the pre-analytical phase has been the focus of sustained quality-improvement efforts in clinical laboratory medicine (16, 17). The cohort-derived center-level percentiles are summarized in Supplementary Table S8 as descriptive lower-tail, quartile, median, and upper-tail references. The 25th, 50th, and 75th percentiles were 0.103, 0.144, and 0.168%, with P10-P90 spanning 0.089–0.195%. These values describe the present cohort and should not be interpreted as universal performance specifications. The approach is conceptually aligned with international quality-indicator benchmarking frameworks for the pre-analytical phase, including IFCC WG-LEPS (18, 19), EFLM WG-PRE (20, 21), and harmonization initiatives (22, 23).

The operational and economic estimates in Table 6 are illustrative and should not be interpreted as financial accounting for the present hospitals. The present study did not include hospital financial data, and published per-event estimates were derived from different healthcare systems, price years, and assumptions about recollection. They are retained only to contextualize the potential resource burden of rejected specimens when recollection is required (7–9).

**Table 6:** Illustrative operational and economic implications of 42,060 venous specimen rejection events based on published per-event estimates.

Reference	Setting	Unit estimate (per rejected specimen requiring recollection)	Cost components (as reported)	Applied count (n)	Implied total (original currency)	Implied total (approx. CNY)	Delay per event	Implied total delay
Eker (7)	Consolidated hospital laboratory system (Turkiye)	€2.10 (2019 prices)	Materials + personnel work-time + logistics/transfer + waste disposal	42,060	€88,326	≈CNY 0.72 million	—	—
Hjelmgren et al. (8)	Tertiary pediatric hospital care (Sweden)	€25.4	Personnel + materials + laboratory analyses + premises/hospitalization	42,060	€1,068,324	≈CNY 8.65 million	—	—
Cao et al. (9)	Large US clinical chemistry laboratory	$21.9	Not fully available in accessible text	42,060	$921,114	≈CNY 6.72 million	108 min	4,542,480 min (75,708 h)

CNY totals are approximate conversions using reference exchange rates in January 2026 (approximately EUR 1 ≈ CNY 8.1; USD 1 ≈ CNY 7.3; exact rates not locked as these figures are order-of-magnitude estimates only). Unit-cost estimates were derived from different healthcare systems and price years (2016–2023), and not all rejected specimens necessarily undergo recollection; therefore, these figures should not be interpreted as precise financial accounting for the present study setting.

After accreditation re-verification, hospital-grade strata were too sparse for stable inferential comparison. We therefore avoided over-interpreting hospital grade as an explanatory variable and instead emphasized residual center-level heterogeneity. The observed ICC (0.045) indicates a modest but detectable center-specific component beyond calendar-month variation, reinforcing the need for benchmark reporting that quantifies heterogeneity rather than relying solely on pooled summaries.

Hemolysis, as a recognized pre-analytical quality challenge affecting laboratory result validity (24), was among the top rejection reason categories and showed substantial between-center variation in relative frequency, reinforcing the importance of standardized documentation across sites. More broadly, standardized quality indicators support the consistent identification and monitoring of errors across the pre-analytical phase (25, 26).

Department-level rate estimates are available only for the seven centers that passed denominator coverage QC (Supplementary Table S10; Supplementary Data S1); excluded centers included high-volume hospitals, limiting generalizability.

### Limitations

1. Department denominators were inconsistent with center venous_total in some centers, limiting interpretability of department-level rejection rates. To mitigate this, department-level rejection rates were reported only for centers passing a prespecified denominator coverage QC (coverage_ratio within [0.98, 1.02]); this necessarily limits the generalizability of department-level rate findings, particularly when excluded centers include high-volume hospitals.
2. Centers may differ in operational definitions and recording workflows for rejection and pre-analytical defects (e.g., instrument-index vs. visual criteria for hemolysis, differences in what constitutes a reportable rejection event, and whether all rejections are captured in the same LIS module). Such heterogeneity may introduce measurement bias when comparing centers.
3. Event logs may contain repeated entries for the same specimen when unique identifiers (barcode/specimen ID) are unavailable or not consistently exported across centers. A retrospective check using a conservative deduplication criterion (identical center, date, department, specimen type, tube type, and rejection reason) identified 6,866 records (16.32% of main analysis events) that matched at least one other record across all fields; while many of these likely represent legitimate multiple rejection events with identical characteristics occurring on the same day in high-volume centers, the possibility that some reflect duplicate log entries cannot be excluded. After strict removal, the pooled rejection rate decreased from 0.197 to 0.165%; the top-3 standardized rejection reasons and their ranking were unchanged (Clot/Aggregation, Insufficient/Incorrect Volume, Container/Tube Error), supporting robustness of the main compositional and ranking analyses even under this conservative scenario.
4. The extremely low rejection rate observed in certain centers may reflect true high performance but may also reflect under-reporting or selective capture of rejection events. This potential information bias cannot be fully excluded without external audit; sensitivity analyses excluding influential centers are recommended.
5. Tube-specific and specimen-specific denominators were unavailable; tube/specimen analyses are therefore composition-based rather than rate-based.
6. Encounter-setting subgroup analyses relied on explicit labels embedded in department names rather than a dedicated structured patient-type field. As a result, only 37.5% of venous rejection events could be conservatively assigned to inpatient, outpatient, emergency, or physical examination settings, and these subgroup summaries should be interpreted as descriptive rather than exhaustive.
7. Specialty-level department standardization was rule-based rather than derived from a uniform regional master dictionary. Although 96.6% of events could be classified after iterative rule expansion, a small residual fraction remained under generic or ambiguous labels, and some local department names may still be grouped imperfectly.
8. After final accreditation re-verification, hospital-grade strata were sparse (3A, *n* = 8 centers; 3B, *n* = 1; 2A, *n* = 1), precluding robust causal interpretation of hospital-grade effects.
9. Centers differed in time coverage, and absolute pooled rates and lower-tail benchmark percentiles were sensitive to the common-month restriction, exclusion of the highest-volume center, and strict duplicate-pattern removal. The percentile values should be treated as descriptive internal references pending external validation.

## Conclusion

A reproducible, denominator-consistent benchmarking framework based on center-level distributions and a prespecified venous_total denominator can support actionable quality management of venous blood specimen rejection. Quantifying between-center heterogeneity and standardizing rejection reasons enhance the transferability of benchmarks to healthcare systems with heterogeneous resources. Stratified descriptive analyses across conservative encounter-setting subsets and standardized specialty departments can further localize intervention priorities, but stronger structured patient-type fields and harmonized department denominators are needed to strengthen future stratified inference.

## Data Availability

The original contributions presented in the study are included in the article/Supplementary material, further inquiries can be directed to the corresponding authors.
