# Supplementary material for: Establishing denominator-consistent Six Sigma benchmarks for venous blood specimen rejection: a multi-center retrospective quality-indicator study using 21 million specimens in Western China
Source: Front Med (Lausanne). 2026 Aug 17;13:1878028. doi: 10.3389/fmed.2026.1878028 (PMC13528587; doi:10.3389/fmed.2026.1878028)
Supplement: Supplementary file 1 [file Supplementary_file_1.docx]

# Supplementary Tables

## Table S1. Standardized Rejection Reason Dictionary

**Table S1. Frozen mapping dictionary for rejection reason standardization.** Each row represents a unique local rejection-reason label observed across centers, shown here in English translation together with its normalized translated form and standardized primary category. The dictionary was frozen prior to quantitative analysis. Entries mapped to "Contamination (Blood Culture)" are excluded from the main analysis and reported separately in Table S2 and Table S3.

| **Original local label (translated)** | **Normalized local label (translated)** | **Standardized Category** | **Count (*n*)** | **Centers** |
| --- | --- | --- | --- | --- |
| Incorrect specimen volume | Incorrect/insufficient collection volume | Insufficient/Incorrect Volume | 8,053 | 004, 035, CB, LS, YA |
| Anticoagulated specimen clotting | Anticoagulant-related clot/aggregation | Clot/Aggregation | 7,245 | 004, 018, LS, YA |
| Incorrect specimen container | Incorrect container/tube | Container/Tube Error | 6,092 | 004, 005, 018, 031, LS, YA, ZG |
| Order error | Order error | Order Error | 3,643 | 004, 005, 018, 030, YA, ZG |
| Other | Other | Other/Unknown | 2,119 | 005, 018, 030, CB, LS, YA, ZG |
| Noncompliant specimen label | Label/identification error | Label/Identification Error | 1,869 | 004, CB |
| Incorrect collection timing | Incorrect collection timing | Timing Error | 1,862 | 004, 031, 035, LS, ZG |
| Clot or aggregation present | Aggregation/clot | Clot/Aggregation | 1,792 | 005 |
| Hemolysis | Hemolysis | Hemolysis | 1,293 | 005 |
| Specimen aggregation | Aggregation/clot | Clot/Aggregation | 918 | 031, ZG |
| Hemolyzed specimen | Hemolyzed specimen | Hemolysis | 906 | 004, 018, 030, 031, CB, LS, YA, ZG |
| Incorrect specimen type | Incorrect specimen type | Specimen Type Error | 858 | 004, 005, 018, 030, 031, 035, LS, YA, ZG |
| Incorrect sample volume | Incorrect/insufficient collection volume | Insufficient/Incorrect Volume | 712 | 005 |
| Anticoagulated specimen clotting rate | Anticoagulant-related clot/aggregation | Clot/Aggregation | 711 | CB |
| Specimen clotting | Aggregation/clot | Clot/Aggregation | 678 | 030 |
| Specimen type error rate | Incorrect specimen type | Specimen Type Error | 471 | CB |
| Inappropriate specimen transport time | Inappropriate transport time | Transport Time | 442 | 004, 018, 031, LS, ZG |
| Incorrect specimen collection volume | Incorrect/insufficient collection volume | Insufficient/Incorrect Volume | 407 | 018, ZG |
| Nonconforming collection volume | Incorrect/insufficient collection volume | Insufficient/Incorrect Volume | 387 | 030 |
| Noncompliant label | Label/identification error | Label/Identification Error | 346 | 031, 035 |
| Excessive specimen transport time rate | Inappropriate transport time | Transport Time | 312 | CB |
| Incorrect collection timing rate | Incorrect collection timing | Timing Error | 312 | CB |
| Duplicate order | Order error | Order Error | 282 | 031 |
| Other (specify in remarks) | Other (specify in remarks) | Other/Unknown | 261 | 004, 031, 035 |
| Container error | Incorrect container/tube | Container/Tube Error | 261 | 030 |
| Insufficient specimen volume | Incorrect/insufficient collection volume | Insufficient/Incorrect Volume | 253 | 031 |
| Excessive submission time | Inappropriate transport time | Transport Time | 134 | 005 |
| Specimen clotting. | Aggregation/clot | Clot/Aggregation | 62 | 035 |
| Specimen identification error | Label/identification error | Label/Identification Error | 53 | 030, YA |
| Severe hemolysis. | Hemolysis | Hemolysis | 41 | 035 |
| Label error | Label/identification error | Label/Identification Error | 39 | 018, ZG |
| Incorrect specimen collection timing | Incorrect collection timing | Timing Error | 22 | 030 |
| Incorrect collection timing | Incorrect collection timing | Timing Error | 10 | 018 |
| Inappropriate transport temperature | Inappropriate transport temperature | Transport Temperature | 7 | 031 |
| Specimen label error | Specimen label/identification error | Label/Identification Error | 7 | LS |
| Inappropriate specimen transport temperature | Inappropriate specimen transport temperature | Transport Temperature | 3 | 004 |
| Excessive specimen submission time | Inappropriate transport time | Transport Time | 2 | 030, YA |
| Incorrect collection container | Incorrect collection container/tube | Container/Tube Error | 2 | 035 |
| Order mismatch | Order error | Order Error | 1 | 035 |
| Blood culture contamination rate | Blood culture contamination | Contamination (Blood Culture)† | 83 | CB |
| Blood culture contamination | Blood culture contamination | Contamination (Blood Culture)† | 19 | 004, ZG |

*n*, frequency count across all centers during the full study window (2023-01-01 to 2024-04-30). Centers identified by study ID (see main text Table 1 for hospital details). †Entries mapped to "Contamination (Blood Culture)" were excluded from the main analysis and are summarized in Table S2 and Table S3.

## Table S2. Blood Culture Contamination Events by Center

**Table S2. Distribution of blood culture contamination events excluded from the main analysis by center.** Blood culture contamination events were prespecified as a separate supplementary dataset and excluded from the main venous blood rejection analysis because blood culture contamination is defined and managed differently from routine venous specimen rejection.

| **Center** | **Hospital Level** | **Blood Culture Events (*n*)** | % of blood culture contamination events |
| --- | --- | --- | --- |
| CB | Tertiary grade A (3A) | 83 | 81.4% |
| ZG | Tertiary grade A (3A) | 15 | 14.7% |
| 004 | Tertiary grade A (3A) | 4 | 3.9% |
| All others | — | 0 | 0.0% |
| Total |  | 102 | 100.0% |

## Table S3. Blood Culture Contamination Reason Distribution

**Table S3. Distribution of local blood culture contamination reason labels excluded from the main analysis.** Local labels are shown in English translation to maintain an all-English appendix. All blood culture contamination events were recorded under contamination-related labels and are presented here as a supplementary descriptive summary.

| **Original local blood-culture label (translated)** | **Standardized Category** | ***n*** | **%** |
| --- | --- | --- | --- |
| Blood culture contamination rate | Contamination (Blood Culture) | 83 | 81.4% |
| Blood culture contamination | Contamination (Blood Culture) | 19 | 18.6% |
| Total |  | 102 | 100.0% |

Blood culture events constituted 0.24% (102/42,970) of all identified rejection records in the study window. Denominator data for blood culture-specific rates were not available for center 004; center CB blood culture rate was approximately 0.190% (83/43,853 blood culture specimens). These results are provided for completeness and should not be combined with the main venous rejection analysis.

## Table S4. Sensitivity Analyses for Benchmarking (Primary Scenarios)

**Table S4. Summary statistics for center-level venous specimen rejection rate under primary sensitivity scenarios.** Full window: 2023-01-01 to 2024-04-30 (16 months). Common months: 2023-05-01 to 2024-04-30 (12 months common across all 10 centers). Excluding center 035 (lowest observed rate) tests robustness of the benchmark distribution.

| **Dataset** | **Excluded Center** | ***N* Centers** | **Pooled Rate (%)** | **Median (%)** | **P25 (%)** | **P75 (%)** | **P10 (%)** | **P90 (%)** |
| --- | --- | --- | --- | --- | --- | --- | --- | --- |
| Full window | Not applicable | 10 | 0.197 | 0.144 | 0.103 | 0.168 | 0.089 | 0.195 |
| Common months | Not applicable | 10 | 0.192 | 0.137 | 0.101 | 0.171 | 0.067 | 0.193 |
| Full window | Center 035 | 9 | 0.199 | 0.147 | 0.128 | 0.174 | 0.094 | 0.207 |
| Common months | Center 035 | 9 | 0.194 | 0.144 | 0.119 | 0.180 | 0.089 | 0.205 |

Pooled rate: volume-weighted (ΣD/ΣN). Percentile statistics (P10, P25, P50, P75, P90) are computed across center-level rejection rates within each scenario. Common months window was defined as the 12-month period with complete data across all 10 centers.

## Table S5. Leave-one-center-out Sensitivity Analysis

**Table S5. Rejection rate benchmark distribution after sequentially excluding each center.** Each row represents the benchmark distribution recomputed after removing one center from the analysis. Results are shown for both the full study window and the common months window.

| **Dataset** | **Excluded Center** | ***N* Centers** | **Pooled Rate (%)** | **Median (%)** | **P25 (%)** | **P75 (%)** | **P10 (%)** | **P90 (%)** |
| --- | --- | --- | --- | --- | --- | --- | --- | --- |
| Full window | 004 | 9 | 0.138 | 0.142 | 0.094 | 0.148 | 0.086 | 0.176 |
| Full window | 005 | 9 | 0.206 | 0.142 | 0.094 | 0.174 | 0.086 | 0.207 |
| Full window | 018 | 9 | 0.200 | 0.147 | 0.094 | 0.174 | 0.086 | 0.207 |
| Full window | 030 | 9 | 0.199 | 0.142 | 0.094 | 0.148 | 0.086 | 0.207 |
| Full window | 031 | 9 | 0.198 | 0.142 | 0.094 | 0.148 | 0.086 | 0.199 |
| Full window | 035 | 9 | 0.199 | 0.147 | 0.128 | 0.174 | 0.094 | 0.207 |
| Full window | CB | 9 | 0.204 | 0.142 | 0.094 | 0.174 | 0.086 | 0.207 |
| Full window | LS | 9 | 0.202 | 0.147 | 0.094 | 0.174 | 0.086 | 0.207 |
| Full window | YA | 9 | 0.204 | 0.147 | 0.128 | 0.174 | 0.089 | 0.207 |
| Full window | ZG | 9 | 0.206 | 0.147 | 0.128 | 0.174 | 0.086 | 0.207 |
| Common months | 004 | 9 | 0.132 | 0.131 | 0.094 | 0.145 | 0.066 | 0.180 |
| Common months | 005 | 9 | 0.200 | 0.131 | 0.094 | 0.180 | 0.066 | 0.205 |
| Common months | 018 | 9 | 0.195 | 0.144 | 0.094 | 0.180 | 0.066 | 0.205 |
| Common months | 030 | 9 | 0.193 | 0.131 | 0.094 | 0.145 | 0.066 | 0.205 |
| Common months | 031 | 9 | 0.193 | 0.131 | 0.094 | 0.145 | 0.066 | 0.204 |
| Common months | 035 | 9 | 0.194 | 0.144 | 0.119 | 0.180 | 0.089 | 0.205 |
| Common months | CB | 9 | 0.199 | 0.131 | 0.094 | 0.180 | 0.066 | 0.205 |
| Common months | LS | 9 | 0.196 | 0.144 | 0.094 | 0.180 | 0.066 | 0.205 |
| Common months | YA | 9 | 0.200 | 0.144 | 0.119 | 0.180 | 0.088 | 0.205 |
| Common months | ZG | 9 | 0.203 | 0.144 | 0.119 | 0.180 | 0.066 | 0.205 |

The most influential center is 004 (largest volume, highest rate): its exclusion reduces the pooled rate from 0.197% to 0.138% (full window) and from 0.192% to 0.132% (common months), illustrating the dominance of volume-weighted estimates by high-volume centers. Percentile-based distributions (median, IQR) are substantially more stable across exclusion scenarios.

## Table S6. Rejection Reason Spectrum Stability Metrics

**Table S6. Pooled rejection-reason ranking after exclusion of Other/Unknown. Because deleting one category preserves the relative order of the remaining categories, these metrics describe pooled reason-set agreement and are not a test of center-level Top-2 stability; the targeted center-level analysis is reported in Table S11.**

| **Metric** | **Value** | **Description** |
| --- | --- | --- |
| Jaccard similarity (Top 3) | 1.000 | Proportion of overlap in the top-3 reason set between both rankings |
| Jaccard similarity (Top 5) | 0.667 | Proportion of overlap in the top-5 reason set between both rankings |
| Jaccard similarity (Top 10) | 0.818 | Proportion of overlap in the top-10 reason set between both rankings |
| Spearman ρ (rank order, shared reasons) | 1.000 | Rank correlation for reasons present in both lists |
| Spearman ρ (% share, shared reasons) | 1.000 | Correlation of percentage shares for shared reasons |
| Shared reason categories (*n*) | 10 | Number of categories present in both comparison lists |

The pooled Top-3 reason set (Clot/Aggregation, Insufficient/Incorrect Volume, and Container/Tube Error) was unchanged after excluding Other/Unknown. This result supports pooled compositional consistency only; it does not establish stability of center-level Pareto concentration.

## Table S7. Full Center-Specific Standardized Reason Distribution

| **center_id** | **reason_std_primary** | **n** | Within-center percentage | **center_total** |
| --- | --- | --- | --- | --- |
| 004 | Insufficient/Incorrect Volume | 6761 | 28.87% | 23421 |
| 004 | Clot/Aggregation | 5315 | 22.69% | 23421 |
| 004 | Container/Tube Error | 5015 | 21.41% | 23421 |
| 004 | Order Error | 2829 | 12.08% | 23421 |
| 004 | Timing Error | 1816 | 7.75% | 23421 |
| 004 | Label/Identification Error | 637 | 2.72% | 23421 |
| 004 | Specimen Type Error | 405 | 1.73% | 23421 |
| 004 | Transport Time | 352 | 1.50% | 23421 |
| 004 | Hemolysis | 279 | 1.19% | 23421 |
| 004 | Other/Unknown | 9 | 0.04% | 23421 |
| 004 | Transport Temperature | 3 | 0.01% | 23421 |
| 005 | Clot/Aggregation | 1783 | 39.64% | 4498 |
| 005 | Hemolysis | 1263 | 28.08% | 4498 |
| 005 | Insufficient/Incorrect Volume | 551 | 12.25% | 4498 |
| 005 | Container/Tube Error | 269 | 5.98% | 4498 |
| 005 | Other/Unknown | 255 | 5.67% | 4498 |
| 005 | Order Error | 238 | 5.29% | 4498 |
| 005 | Transport Time | 75 | 1.67% | 4498 |
| 005 | Specimen Type Error | 64 | 1.42% | 4498 |
| 018 | Other/Unknown | 409 | 31.37% | 1304 |
| 018 | Clot/Aggregation | 347 | 26.61% | 1304 |
| 018 | Insufficient/Incorrect Volume | 176 | 13.50% | 1304 |
| 018 | Container/Tube Error | 147 | 11.27% | 1304 |
| 018 | Hemolysis | 124 | 9.51% | 1304 |
| 018 | Order Error | 42 | 3.22% | 1304 |
| 018 | Label/Identification Error | 23 | 1.76% | 1304 |
| 018 | Specimen Type Error | 18 | 1.38% | 1304 |
| 018 | Timing Error | 10 | 0.77% | 1304 |
| 018 | Transport Time | 8 | 0.61% | 1304 |
| 030 | Other/Unknown | 851 | 31.28% | 2721 |
| 030 | Clot/Aggregation | 678 | 24.92% | 2721 |
| 030 | Insufficient/Incorrect Volume | 387 | 14.22% | 2721 |
| 030 | Order Error | 309 | 11.36% | 2721 |
| 030 | Container/Tube Error | 261 | 9.59% | 2721 |
| 030 | Hemolysis | 145 | 5.33% | 2721 |
| 030 | Label/Identification Error | 51 | 1.87% | 2721 |
| 030 | Timing Error | 22 | 0.81% | 2721 |
| 030 | Specimen Type Error | 16 | 0.59% | 2721 |
| 030 | Transport Time | 1 | 0.04% | 2721 |
| 031 | Label/Identification Error | 343 | 20.21% | 1697 |
| 031 | Order Error | 276 | 16.26% | 1697 |
| 031 | Insufficient/Incorrect Volume | 251 | 14.79% | 1697 |
| 031 | Clot/Aggregation | 245 | 14.44% | 1697 |
| 031 | Other/Unknown | 210 | 12.37% | 1697 |
| 031 | Hemolysis | 130 | 7.66% | 1697 |
| 031 | Container/Tube Error | 99 | 5.83% | 1697 |
| 031 | Specimen Type Error | 59 | 3.48% | 1697 |
| 031 | Transport Time | 59 | 3.48% | 1697 |
| 031 | Timing Error | 18 | 1.06% | 1697 |
| 031 | Transport Temperature | 7 | 0.41% | 1697 |
| 035 | Clot/Aggregation | 62 | 37.58% | 165 |
| 035 | Hemolysis | 41 | 24.85% | 165 |
| 035 | Other/Unknown | 37 | 22.42% | 165 |
| 035 | Specimen Type Error | 15 | 9.09% | 165 |
| 035 | Insufficient/Incorrect Volume | 5 | 3.03% | 165 |
| 035 | Container/Tube Error | 2 | 1.21% | 165 |
| 035 | Label/Identification Error | 1 | 0.61% | 165 |
| 035 | Order Error | 1 | 0.61% | 165 |
| 035 | Timing Error | 1 | 0.61% | 165 |
| CB | Label/Identification Error | 1184 | 29.64% | 3995 |
| CB | Insufficient/Incorrect Volume | 823 | 20.60% | 3995 |
| CB | Clot/Aggregation | 711 | 17.80% | 3995 |
| CB | Specimen Type Error | 471 | 11.79% | 3995 |
| CB | Timing Error | 309 | 7.73% | 3995 |
| CB | Transport Time | 308 | 7.71% | 3995 |
| CB | Hemolysis | 122 | 3.05% | 3995 |
| CB | Other/Unknown | 67 | 1.68% | 3995 |
| LS | Clot/Aggregation | 888 | 55.43% | 1602 |
| LS | Other/Unknown | 257 | 16.04% | 1602 |
| LS | Insufficient/Incorrect Volume | 248 | 15.48% | 1602 |
| LS | Container/Tube Error | 95 | 5.93% | 1602 |
| LS | Hemolysis | 56 | 3.50% | 1602 |
| LS | Specimen Type Error | 18 | 1.12% | 1602 |
| LS | Timing Error | 17 | 1.06% | 1602 |
| LS | Transport Time | 16 | 1.00% | 1602 |
| LS | Label/Identification Error | 7 | 0.44% | 1602 |
| YA | Clot/Aggregation | 621 | 56.56% | 1098 |
| YA | Container/Tube Error | 181 | 16.48% | 1098 |
| YA | Other/Unknown | 130 | 11.84% | 1098 |
| YA | Insufficient/Incorrect Volume | 117 | 10.66% | 1098 |
| YA | Order Error | 17 | 1.55% | 1098 |
| YA | Specimen Type Error | 16 | 1.46% | 1098 |
| YA | Hemolysis | 13 | 1.18% | 1098 |
| YA | Label/Identification Error | 2 | 0.18% | 1098 |
| YA | Transport Time | 1 | 0.09% | 1098 |
| ZG | Clot/Aggregation | 672 | 43.10% | 1559 |
| ZG | Specimen Type Error | 234 | 15.01% | 1559 |
| ZG | Insufficient/Incorrect Volume | 229 | 14.69% | 1559 |
| ZG | Container/Tube Error | 223 | 14.30% | 1559 |
| ZG | Other/Unknown | 100 | 6.41% | 1559 |
| ZG | Order Error | 46 | 2.95% | 1559 |
| ZG | Hemolysis | 35 | 2.25% | 1559 |
| ZG | Label/Identification Error | 14 | 0.90% | 1559 |
| ZG | Timing Error | 5 | 0.32% | 1559 |
| ZG | Transport Time | 1 | 0.06% | 1559 |

## Table S8. Cohort-derived center-level rejection-rate percentiles

**Table S8. Cohort-derived center-level rejection-rate percentiles. These empirical percentiles are descriptive internal references and are not externally validated or universal performance specifications.**

| Empirical reference | **Percentile** | **Rejection Rate (%)** | **Interpretation** |
| --- | --- | --- | --- |
| P10 | 10th | 0.089 | Lower-tail empirical reference |
| P25 | 25th | 0.103 | Lower quartile |
| P50 | 50th | 0.144 | Cohort median |
| P75 | 75th | 0.168 | Upper quartile |
| P90 | 90th | 0.195 | Upper-tail empirical reference |

Percentiles were computed from 10 center-level rejection rates over the full study window. Common-month, influential-center, and strict duplicate-pattern sensitivity results are reported in Tables S4, S5, and S14 and should be considered when interpreting absolute thresholds.

## Table S9. Encounter-setting sample sizes and leading rejection reasons

The analysis is restricted to the 15,757 events with explicit encounter-setting labels; 26,303 events without an explicit setting label remain unclassified and are not redistributed. Percentages are within each eligible setting.

| **Encounter setting** | **Eligible events (n)** | **Rank** | **Standardized reason** | **Reason events (n)** | **Within-setting (%)** |
| --- | --- | --- | --- | --- | --- |
| Inpatient | 8483 | 1 | Clot/Aggregation | 3033 | 35.75 |
| Inpatient | 8483 | 2 | Insufficient/Incorrect Volume | 2056 | 24.24 |
| Inpatient | 8483 | 3 | Container/Tube Error | 775 | 9.14 |
| Emergency | 2392 | 1 | Clot/Aggregation | 679 | 28.39 |
| Emergency | 2392 | 2 | Insufficient/Incorrect Volume | 488 | 20.40 |
| Emergency | 2392 | 3 | Container/Tube Error | 313 | 13.09 |
| Outpatient | 4491 | 1 | Insufficient/Incorrect Volume | 1148 | 25.56 |
| Outpatient | 4491 | 2 | Order Error | 858 | 19.10 |
| Outpatient | 4491 | 3 | Timing Error | 657 | 14.63 |
| Physical examination | 391 | 1 | Label/Identification Error | 144 | 36.83 |
| Physical examination | 391 | 2 | Order Error | 51 | 13.04 |
| Physical examination | 391 | 3 | Clot/Aggregation | 47 | 12.02 |

## Table S10. Department-denominator coverage QC and availability of department-level rejection rates

Department-level rates were retained only when the center's summed department denominator divided by center venous_total was within 0.98-1.02. Full passing-center department estimates with Wilson 95% confidence intervals are supplied in Supplementary Data S1.

| **Center** | **Center venous_total** | **Sum of department denominators** | **Coverage ratio** | **QC status** | **Departments with rate estimates (n)** | **Minimum rate (%)** | **Median rate (%)** | **Maximum rate (%)** |
| --- | --- | --- | --- | --- | --- | --- | --- | --- |
| 004 | 7806713 | 9741171 | 1.2478 | Excluded | 0 | Not applicable | Not applicable | Not applicable |
| 005 | 3062426 | 3062426 | 1.0000 | Pass | 64 | 0.000 | 0.149 | 0.885 |
| 018 | 918396 | 918379 | 1.0000 | Pass | 66 | 0.000 | 0.018 | 1.348 |
| 030 | 1561199 | 1561199 | 1.0000 | Pass | 79 | 0.000 | 0.061 | 12.500 |
| 031 | 926318 | 900572 | 0.9722 | Excluded | 0 | Not applicable | Not applicable | Not applicable |
| 035 | 244149 | 244149 | 1.0000 | Pass | 24 | 0.000 | 0.037 | 0.208 |
| CB | 2692558 | 2692559 | 1.0000 | Pass | 158 | 0.000 | 0.000 | 1.038 |
| LS | 1250185 | 1250372 | 1.0001 | Pass | 112 | 0.000 | 0.000 | 1.970 |
| YA | 1204237 | 1204237 | 1.0000 | Pass | 103 | 0.000 | 0.000 | 3.030 |
| ZG | 1653019 | 2010794 | 1.2164 | Excluded | 0 | Not applicable | Not applicable | Not applicable |

## Table S11. Center-level Top-2 sensitivity after excluding Other/Unknown

The original analysis includes Other/Unknown when it is among a center's two most frequent categories. The sensitivity analysis removes Other/Unknown from both the ranking and denominator before recalculating the two leading known-category shares.

| **Center** | **Other/Unknown (n)** | **Other/Unknown (%)** | **Original Top-2 categories** | **Original Top-2 share (%)** | **Top-2 categories excluding Other/Unknown** | **Top-2 share excluding Other/Unknown (%)** | **Absolute change (percentage points)** |
| --- | --- | --- | --- | --- | --- | --- | --- |
| 004 | 9 | 0.04 | Insufficient/Incorrect Volume; Clot/Aggregation | 51.56 | Insufficient/Incorrect Volume; Clot/Aggregation | 51.58 | +0.02 |
| 005 | 255 | 5.67 | Clot/Aggregation; Hemolysis | 67.72 | Clot/Aggregation; Hemolysis | 71.79 | +4.07 |
| 018 | 409 | 31.37 | Other/Unknown; Clot/Aggregation | 57.98 | Clot/Aggregation; Insufficient/Incorrect Volume | 58.44 | +0.46 |
| 030 | 851 | 31.28 | Other/Unknown; Clot/Aggregation | 56.19 | Clot/Aggregation; Insufficient/Incorrect Volume | 56.95 | +0.76 |
| 031 | 210 | 12.37 | Label/Identification Error; Order Error | 36.48 | Label/Identification Error; Order Error | 41.63 | +5.15 |
| 035 | 37 | 22.42 | Clot/Aggregation; Hemolysis | 62.42 | Clot/Aggregation; Hemolysis | 80.47 | +18.04 |
| CB | 67 | 1.68 | Label/Identification Error; Insufficient/Incorrect Volume | 50.24 | Label/Identification Error; Insufficient/Incorrect Volume | 51.09 | +0.86 |
| LS | 257 | 16.04 | Clot/Aggregation; Other/Unknown | 71.47 | Clot/Aggregation; Insufficient/Incorrect Volume | 84.46 | +12.99 |
| YA | 130 | 11.84 | Clot/Aggregation; Container/Tube Error | 73.04 | Clot/Aggregation; Container/Tube Error | 82.85 | +9.81 |
| ZG | 100 | 6.41 | Clot/Aggregation; Specimen Type Error | 58.11 | Clot/Aggregation; Specimen Type Error | 62.10 | +3.98 |

## Table S12. Center-level distribution of locally recorded tube-label proxy groups

Additive information was not uniformly available. Color-derived groups are standardized local proxy labels observed among rejection events and should not be interpreted as verified additive-specific tube classes or tube-specific rejection rates.

| **Center** | **Locally recorded tube-label proxy group** | **Rejection events (n)** | **Within-center tube-labeled events (%)** |
| --- | --- | --- | --- |
| 004 | Purple label | 7071 | 30.19 |
| 004 | Blue label | 7001 | 29.89 |
| 004 | Red label | 6957 | 29.70 |
| 004 | Green label | 2179 | 9.30 |
| 004 | Gray label | 186 | 0.79 |
| 004 | Other/Unknown label | 27 | 0.12 |
| 005 | Purple label | 1718 | 38.19 |
| 005 | Yellow label | 1560 | 34.68 |
| 005 | Blue label | 1079 | 23.99 |
| 005 | Other/Unknown label | 100 | 2.22 |
| 005 | Green label | 41 | 0.91 |
| 018 | Purple label | 634 | 48.62 |
| 018 | Red label | 351 | 26.92 |
| 018 | Blue label | 182 | 13.96 |
| 018 | Green label | 101 | 7.75 |
| 018 | Yellow label | 36 | 2.76 |
| 030 | Purple label | 813 | 29.88 |
| 030 | Blue label | 792 | 29.11 |
| 030 | Red label | 637 | 23.41 |
| 030 | Other/Unknown label | 393 | 14.44 |
| 030 | Green label | 85 | 3.12 |
| 030 | Yellow label | 1 | 0.04 |
| 031 | Purple label | 585 | 34.47 |
| 031 | Red label | 561 | 33.06 |
| 031 | Blue label | 313 | 18.44 |
| 031 | Other/Unknown label | 177 | 10.43 |
| 031 | Green label | 61 | 3.59 |
| 035 | Yellow label | 72 | 43.64 |
| 035 | Purple label | 52 | 31.52 |
| 035 | Blue label | 41 | 24.85 |
| CB | Purple label | 1189 | 29.76 |
| CB | Green label | 1137 | 28.46 |
| CB | Blue label | 969 | 24.26 |
| CB | Red label | 668 | 16.72 |
| CB | Other/Unknown label | 32 | 0.80 |
| LS | Purple label | 531 | 33.15 |
| LS | Other/Unknown label | 370 | 23.10 |
| LS | Blue label | 365 | 22.78 |
| LS | Yellow label | 282 | 17.60 |
| LS | Green label | 48 | 3.00 |
| LS | Red label | 6 | 0.37 |
| YA | Purple label | 466 | 42.44 |
| YA | Blue label | 335 | 30.51 |
| YA | Yellow label | 207 | 18.85 |
| YA | Other/Unknown label | 76 | 6.92 |
| YA | Green label | 14 | 1.28 |
| ZG | Purple label | 643 | 41.24 |
| ZG | Yellow label | 334 | 21.42 |
| ZG | Blue label | 325 | 20.85 |
| ZG | Other/Unknown label | 189 | 12.12 |
| ZG | Gray label | 49 | 3.14 |
| ZG | Red label | 13 | 0.83 |
| ZG | Green label | 6 | 0.38 |

## Table S13. Center-level extraction-field and denominator quality-control summary

No common LIS query string was applicable across heterogeneous systems. Each source export was audited for the prespecified six event fields (date, specimen type, tube label, department, rejection reason, and test item) and matching monthly denominators. Rates were not extrapolated beyond submitted months.

| **Center** | **Event fields present** | **Source specimen-field label** | **Monthly denominator fields present** | **Submitted denominator months (n)** | **Denominator QC flags (nonpositive / total<venous / venous<anticoag)** | **Department denominator coverage ratio** | **Department-rate QC** |
| --- | --- | --- | --- | --- | --- | --- | --- |
| 004 | 6/6 | Sample type | Yes | 16 | 0 / 0 / 0 | 1.2478 | Excluded |
| 005 | 6/6 | Sample type | Yes | 16 | 0 / 0 / 0 | 1.0000 | Pass |
| 018 | 6/6 | Sample type | Yes | 16 | 0 / 0 / 0 | 1.0000 | Pass |
| 030 | 6/6 | Sample type | Yes | 16 | 0 / 0 / 0 | 1.0000 | Pass |
| 031 | 6/6 | Sample type | Yes | 16 | 0 / 0 / 0 | 0.9722 | Excluded |
| 035 | 6/6 | Sample type | Yes | 16 | 0 / 0 / 0 | 1.0000 | Pass |
| CB | 6/6 | Sample type | Yes | 16 | 0 / 0 / 0 | 1.0000 | Pass |
| LS | 6/6 | Sample type | Yes | 16 | 0 / 0 / 0 | 1.0001 | Pass |
| YA | 6/6 | Sample type | Yes | 16 | 0 / 0 / 0 | 1.0000 | Pass |
| ZG | 6/6 | Specimen type | Yes | 12 | 0 / 0 / 0 | 1.2164 | Excluded |

## Table S14. Conservative duplicate-pattern sensitivity by center

Potential duplicates were defined as records identical on center, date, department, specimen type, tube label, and rejection reason. Without unique specimen identifiers, this strict rule may remove legitimate same-day events and is therefore a sensitivity analysis rather than a corrected primary dataset.

| **Center** | **Original events (n)** | **Events after strict removal (n)** | **Potential duplicate-pattern records removed (n)** | **Removed (%)** |
| --- | --- | --- | --- | --- |
| 004 | 23421 | 18681 | 4740 | 20.24 |
| 005 | 4498 | 4166 | 332 | 7.38 |
| 018 | 1304 | 1076 | 228 | 17.48 |
| 030 | 2721 | 2099 | 622 | 22.86 |
| 031 | 1697 | 1543 | 154 | 9.07 |
| 035 | 165 | 134 | 31 | 18.79 |
| CB | 3995 | 3465 | 530 | 13.27 |
| LS | 1602 | 1553 | 49 | 3.06 |
| YA | 1098 | 985 | 113 | 10.29 |
| ZG | 1559 | 1492 | 67 | 4.30 |
| Overall | 42060 | 35194 | 6866 | 16.32 |
